# Color Crosstalk Correction in Linear Stokes Imaging Using a Color Polarization Camera with Simultaneous Three Wavelengths Illumination

**DOI:** 10.3390/s26123838

**Published:** 2026-06-16

**Authors:** Manal Altaweel, Judit Bisbal-Amat, Juan Campos, Ángel Lizana, Irene Estévez

**Affiliations:** Group of Optics, Department of Physics, Universitat Autònoma de Barcelona, 08193 Bellaterra, Spain; manal.ibrahimahmad@autonoma.cat (M.A.); judit.bisbal@uab.cat (J.B.-A.); angel.lizana@uab.cat (Á.L.); irene.estevez@uab.cat (I.E.)

**Keywords:** optical polarization, Stokes parameters, polarization cameras

## Abstract

Polarimetric color cameras are a forefront technology that simultaneously captures polarimetric and color information by analyzing polarization states across different color channels, commonly red, green, and blue. In general, each of these color channels can carry different polarization information. Therefore, measuring the polarization Stokes vector at several discrete wavelengths simultaneously and with the highest possible resolution is of interest in multiple research areas. However, when a commercial color polarization sensor is used under simultaneous narrowband RGB illumination mode, its channels cannot be assumed to represent independent wavelength channels. Spectral overlap of the color filters introduces color crosstalk between wavelength-dependent analyzer intensities, which may bias the reconstructed Stokes parameters if it is not corrected before polarimetric inversion. Several methods have been proposed in the literature to address the color crosstalk problem but they typically assume that the polarization state is identical for all wavelengths. This assumption does not generally hold for real samples, which exhibit wavelength-dependent depolarization, retardance, and dichroism. To the best of our knowledge, this is the first work presenting a method that addresses the color crosstalk problem without assuming that the polarization state is identical across all wavelengths. In addition, Fourier domain demosaicking techniques are applied to interpolate the data and reconstruct the images. The present study demonstrates how the proposed method leads to an accurate recovery of chromatic and polarimetric information on both synthetic and real-world datasets. To test our approach, narrowband light beams at three wavelengths (470, 554, 630 nm), with different spatial polarization and degree of linear polarization distributions, have been simulated and validated with simulated and experimental data. The results demonstrate the feasibility of the method for accurate three polarization channels measurements.

## 1. Introduction

Some fundamental physical quantities associated with light are intensity, wavelength, coherence, phase, and polarization. With regard to polarization, polarimetric imaging techniques have become an excellent tool not only for enhanced vision applications, but also for revealing information that remains hidden in other non-polarimetric imaging methods. Applications of polarization imaging include remote sensing [1], astronomy [2], applications in industry [3], biophotonics [4], machine vision [5] and general contrast enhancement [6]. Thus, enhancements to polarimetric systems may significantly contribute to the advancement of the aforementioned fields. In many optical metrology systems, the polarization state is described by the full or partial Stokes vector recorded across the image plane [7]. Accurate Stokes reconstruction is therefore a key step not only in direct polarimetric imaging, but also in Stokes–Mueller polarimetry, where a calibrated polarization state analyzer is required to estimate the Mueller matrix of the sample [8,9]. In polarimetric imaging, the measurement process depends on wavelength, so that polarization information is measured at different spectral bands. In many applications, the polarization contrast may vary from one wavelength to another, so, each wavelength should be treated with its own calibrated analyzer response. In this scenario, the reconstructed Stokes information therefore required wavelength-specific polarization state analyzer response.

Division-of-amplitude polarimetry is one of the established approaches for fast polarimetric metrology, enabling real-time acquisition of the Stokes vector [10,11]. This technique avoids temporal modulation by splitting the incoming beam into multiple optical paths, each analyzed independently. However, it typically requires several optical channels and beam splitters, which increases system complexity, size, and alignment requirements. An alternative to this approach is microgrid or division-of-focal-plane (DoFP) polarimetric imaging, which also enables real-time acquisition. In this case, an array of micro-polarizers is integrated directly onto the sensor plane, allowing different polarization analyzer intensities to be captured in a single snapshot [12,13,14]. Comparing these two approaches, while DoFP inherently reduces spatial resolution due to the multiplexing of polarization information at the pixel level, it enables the implementation of compact, single-path optical systems, in contrast to division-of-amplitude setups that rely on multiple projection arms. DoFP polarimeters are specifically designed to analyze the direction of the light’s electric field oscillation. The emergence of DoFP image sensors represents a significant milestone in the evolution of polarization imaging, providing a versatile and efficient means of capturing polarization information in real time [12,15], without the complexity associated with multi-path optical configurations.

A set of independent polarization sensitive measurements (at least four) are required to obtain a calibrated polarization state analyzer matrix for the full Stokes vector determination of a light beam. Before the arrival of Microgrid Polarimetric Imaging (MPI), this situation was typically achieved by complex setups [16], or time-sequential configurations [17], which made such characterization procedure based on bulky systems or time-consuming, respectively. These limitations prevented the polarization systems from being implemented in real-time applications. However, the emergence of MPI devices enabled the acquisition of spatial information while overcoming these limitations, using sensors consisting of ordered arrays of four-polarization based macro-pixels. Consequently, this novel technology has attracted considerable interest in applications requiring rapid measurement of structures with spatially varying polarization.

**Figure 1:**
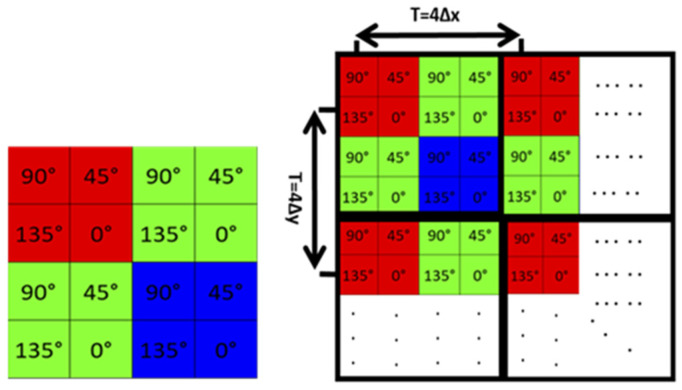
(**left**): The unitary pixel cell (or super pixel). (**right**): The full polarimetric color sensor consists of repeated 12 different analyzers (super pixel) following a periodic pattern. The closer pixel of the same class is found at distances of 4·Δx and 4·Δy. The dots mean that the structure is repeated periodically.

Additionally, a valuable approach for imaging applications is to combine spectroscopic and polarimetric information [18], which can be beneficial for image enhancing and automatic classification purposes. Under this scenario, some authors have presented configurations able to integrate the advantages of DoFP and integral field spectroscopy to implement a linear Stokes imaging spectropolarimeter [19]. Moreover, recent achievements in image sensor manufacturing technology have given rise to novel RGB polarimetric cameras [20,21,22], built with single-chip polarized color sensors, capable of capturing both RGB and polarization data in a mosaic form. The typical architecture of these sensors comprises arrays of super pixels, organized as groups of 4 × 4 pixels, including blocks of four directional polarizers (typically at angles of 0°, 45°, 90° and 135°), each one associated with a different color filter (red, green, green and blue, resulting in RGGB-type polarimetric cameras). The raw mosaic image obtained by this sensor should be first separated into the 12 types of color/polarization analyzers, forming the required RGGB–polarization state analyzers pattern (see an example in the illustration shown Figure 1). This step is essential in Stokes–Mueller polarimetry because the analyzed intensities are governed by the wavelength-dependent response of the system. Under these conditions, by properly processing information provided by polarimetric color cameras, the linear content of the Stokes image associated with a given scene or sample, at three different wavelengths, can be retrieved.

Despite many benefits, there are some limitations to consider. On the one hand, the non-ideality of micro-polarizer sensors’ responses, but for that, calibration methods for DoFP and RGB micro-grid polarimetric cameras have been developed [9,23]. Moreover, polarimetric cameras sacrifice spatial resolution to form the stated super pixels. To overcome this situation, the use of interpolation algorithms becomes mandatory [8,22], and many studies have already focused on color polarization demosaicking including interpolation and edge-aware approaches [12,24], model inversion methods [25], Fourier domain filtering [26], sparse reconstruction [27], and learning-based or Stokes fusion methods [28,29]. These approaches are essential for recovering the spatially undersampled color polarization channels of color polarization filter array sensors; however, their main objective is usually image reconstruction rather than metrological propagation of spectral leakage into wavelength-dependent Stokes measurements. Therefore, demosaicking alone does not solve the problem addressed here, since a reconstructed RGB polarization mosaic may still contain spectrally mixed analyzer intensities. On the other hand, the inclusion of RGGB polarimetry snapshot sensors typically involves color crosstalk between filters, and the RGB color filters responses are spectrally mixed, which can introduce errors in the measurement process, leading to inaccuracies in determining the polarization state of light. In parallel, color polarization sensors have been exploited for snapshot multispectral imaging [30], where the different spectral contributions are mixed in the camera channels and must be separated using crosstalk correction in order to recover wavelength-dependent images. In such systems, the polarization dimension is often exploited as an encoding mechanism to separate spectral information, and crosstalk correction is mainly used to recover wavelength-dependent intensity images. In contrast, in the present work the polarization information is not only an encoding variable, but the physical quantity to be measured. Consequently, residual color crosstalk directly biases the analyzer intensities and propagates into the reconstructed Stokes parameters and the derived polarization quantities. Together, these works demonstrate the known challenges in the broader field of color polarization imaging.

However, fewer works have considered the commercial RGB DOFP polarization camera as a calibrated multi-wavelength polarimetric measurement system. For example, recent snapshots of RGB birefringence imaging have demonstrated that simultaneous RGB-LED illumination combined with a color polarized camera can extract multi-wavelength birefringence information in a single acquisition [31]. Nevertheless, this approach focuses on estimating the retardance and fast axis and does not explicitly formulate color channel unmixing as a correction step prior to Stokes inversion. Similarly, full-Stokes RGB camera architectures have taken into account the color dependence of Bayer filter responses through dedicated hardware and calibration models [16], but they do not address the specific case studied here: correcting the raw RGB channels mixing for a commercial single sensor RGB DoFP polarized camera operating under three simultaneous narrowband illumination sources. Several authors have proposed interesting approaches to address the problem of full Stokes polarimetry at different wavelengths in one shot. For instance, Ref. [32] presents a simple pathway for color polarization vision. However, most of these approaches rely on the assumption that the polarization states of beams at different wavelengths are identical, an assumption that does not generally hold in realistic scenarios.

From this perspective, our goal is to measure accurately the polarimetric response of samples when using three selected nearly monochromatic light sources simultaneously. This distinction is important because in polarimetric metrology, the RGB channels cannot be assumed to carry independent and identical polarization information. The wide band transmission of the Bayer filters produces a crosstalk between the color channels, which can lead to significant errors in the measured analyzer intensities and, therefore, the determination of the Stokes vectors distribution carried out by the three wavelengths, if it is not corrected before the polarimetric inversion.

In this paper, we present a calibrated wavelength-resolved linear polarimetric imaging pipeline using a color division-of-focal-plane polarization camera. The contribution is not a new generic demosaicking algorithm, but the integration of Fourier domain color polarization demosaicking [26,33], experimentally calibrated RGB crosstalk correction, and wavelength-specific Stokes reconstruction into a single framework for simultaneous three-wavelength polarimetric measurement. The method is evaluated in simulation and experiment by comparing reconstructions before and after crosstalk correction for the red, green, and blue channels. Quantitative metrics are reported for DoLP, normalized linear Stokes vector error, AoLP, and inferred El, together with robustness analysis under noise and crosstalk matrix perturbation.

We want to note that other effects may also play a relevant role when calibrating the behavior of polarimetric cameras. For instance, spatial crosstalk between neighboring pixels can influence the measured signals and potentially degrade accuracy. However, the primary focus of this work is on color crosstalk under simultaneous three-wavelength illumination, which, as demonstrated in this study, constitutes a dominant source of error affecting the precision of the measurements. Importantly, our approach does not rely on the assumption of identical polarization states across all wavelengths as other approaches in the literature, thereby making it applicable to a wide range of realistic measurement scenarios.

The structure of this work is organized as follows: Section 2 presents the camera model and the color crosstalk correction formalism. Section 3 presents the reconstruction pipeline, simulations, robustness analysis, and experimental validation. Section 4 discusses the scope, limitations, and applicability of the method.

## 2. Materials and Methods

### 2.1. Color Polarimetric Camera Model

As previously stated, the polarimetric color camera consists of 12 color polarization channels, considering the four different linear polarizations and the three-color filters, with green channel sampled twice within the Bayer pattern. This spatially multiplexed mosaic, including the unit pixel cell, or super pixel, is shown in Figure 1(left). To complete the camera sensor, this super pixel is replicated over the full sensor following a periodic pattern. Under this scheme, if an individual pixel has a size of Δx and Δy in the x and y directions respectively, when considering a particular pixel class, the closer pixel of the same class is found at distances of 4·Δx and 4·Δy respectively (see Figure 1(right)). Consequently, for a camera sensor with (N × N) pixels dimensions, the 12 polarimetric images obtained by using the polarimetric color camera present an N/4 × N/4 dimensions, and thus, present a reduced resolution compared to the camera sensor resolution. Under this scenario, the raw mosaic image must be separated into different color polarization channels and reconstructed at the full image resolution before being used for Stokes calculation purposes. After the interpolation step, 12 (N × N) full resolution intensity images are obtained, each one corresponding to a specific combination of polarizer orientation and a color filter.

### 2.2. Mathematical Formalism of Color Polarimetric Camera Model

The polarimetric response of a sample, such as biological tissue, depends on the illuminating wavelength. Thus, using more than one wavelength to inspect a sample is likely to provide additional information about it. Our aim is to illuminate the samples with three wavelengths simultaneously and measure the polarization responses for all three-color filters. To this end, we use three LEDs with interferential filters that have spectral bandwidths of about 10 nanometers, and central wavelengths of 630 nm, 554 nm, and 470 nm. However, the spectral color band of the Bayer filters in the camera are very broad, causing the information carried out by the different wavelengths to overlap. This leads to color crosstalk effects, where polarization signals from different colors overlap. We propose a method to mitigate this effect and ensure accurate separation and measurement of polarimetric and color information. This section is devoted to providing a method able to reduce the crosstalk effect associated with color polarimetric cameras.

We now introduce a measurement model to address the issue of color crosstalk that occurs between the different color channels in images obtained through the camera. Let ${\vec{S}}^{\lambda }\left(x,y\right),\lambda =\lambda_{1},\lambda_{2},\lambda_{3}$ be the three polarization distributions (Stokes vectors) corresponding to the three wavelengths used. In each pixel, there is one of the four polarization analyzers, corresponding to the orientation of the micro-polarizers. In addition, as mentioned above, each pixel is covered by a different color filter that exhibits some transmission for each of the three wavelengths. Let ($t_{f}^{\lambda }$) be the intensity transmission of a color filter (*f*) for a wavelength (*λ*). Let ${\vec{A}}_{p}^{\lambda }$ be the analyzer corresponding to one of the four-polarizer orientation *p* for a wavelength (*λ*). Then, given a pixel with a polarizer orientation (*p*) in front of a color filter (*f*) illuminated with a wavelength *λ*, the resulting intensity $\left(I_{pf}^{\lambda }\right)$ will be:

(1) $$I_{pf}^{\lambda }=t_{f}^{\lambda }({\vec{A}}_{p}^{\lambda }){\vec{S}}^{\lambda }=t_{f}^{\lambda }I_{p}^{\lambda }.$$

If the three wavelengths work at the same time, the resulting intensity will be:

(2) $$I_{pf}^{ct}=\sum_{\lambda }I_{pf}^{\lambda }=\sum_{\lambda }t_{f}^{\lambda }({\vec{A}}_{p}^{\lambda }){\vec{S}}^{\lambda }=\sum_{\lambda }t_{f}^{\lambda }I_{p}^{\lambda }.$$

Note that $I_{pf}^{ct}$ are the obtained intensities after performing the interpolation in intensity measurements. Moreover, the super-index (*ct*) indicates that these intensities are affected by crosstalk. Therefore, to obtain the three polarization distributions we should be able to get the intensity distributions $I_{p}^{\lambda }$ by solving Equation (2). This equation can be rewritten in a matrix form as follows:

(3) $$I_{pf}^{ct}=CI_{p}^{\lambda },$$

where *C* is the color crosstalk transfer matrix relating $I_{p}^{\lambda }$ with $I_{pf}^{ct}$, $I_{p}^{\lambda }$ being the original intensity distribution corresponding to the polarizer orientation “*p*” and wavelength “*λ*”, and the measured distribution affected by the crosstalk.

To solve (3) the matrix C must be experimentally calibrated. Thus, we have measured the transmission intensities of the three-color filters for our case. In particular, our experiment deals with a polarimetric RGGB camera by Lucid Vision (Lucid Vision Labs Inc., Burnaby, BC, Canada), which is equipped with a Sony IMX250MYR CMOS sensor.

To calculate the transmission intensities of the RGB color filters, we illuminated the camera with one wavelength at a time, using either left or right circularly polarized light. In this way, the intensity transmitted by the camera polarizers does not depend on their orientation. These measurements can also be done with fully unpolarized light. The results are arranged in Table 1.

Considering the experimental transmission intensities given in Table 1, the relation given by (3) can be written in a matrix form as:

(4) $$\left(\begin{array}{c} I_{1,1}^{ct}\\ I_{2,1}^{ct}\\ I_{3,1}^{ct}\\ I_{4,1}^{ct}\\ I_{1,2}^{ct}\\ I_{2,2}^{ct}\\ I_{3,2}^{ct}\\ I_{4,2}^{ct}\\ I_{1,3}^{ct}\\ I_{2,3}^{ct}\\ I_{3,3}^{ct}\\ I_{4,3}^{ct} \end{array}\right)=\left(\begin{array}{cccccccccccc} 1 & 0 & 0 & 0 & 0.04 & 0 & 0 & 0 & 0.01 & 0 & 0 & 0\\ 0 & 1 & 0 & 0 & 0 & 0.04 & 0 & 0 & 0 & 0.01 & 0 & 0\\ 0 & 0 & 1 & 0 & 0 & 0 & 0.04 & 0 & 0 & 0 & 0.01 & 0\\ 0 & 0 & 0 & 1 & 0 & 0 & 0 & 0.04 & 0 & 0 & 0 & 0.01\\ 0.25 & 0 & 0 & 0 & 1 & 0 & 0 & 0 & 0.19 & 0 & 0 & 0\\ 0 & 0.25 & 0 & 0 & 0 & 1 & 0 & 0 & 0 & 0.19 & 0 & 0\\ 0 & 0 & 0.25 & 0 & 0 & 0 & 1 & 0 & 0 & 0 & 0.19 & 0\\ 0 & 0 & 0 & 0.25 & 0 & 0 & 0 & 1 & 0 & 0 & 0 & 0.19\\ 0.03 & 0 & 0 & 0 & 0.1 & 0 & 0 & 0 & 0.72 & 0 & 0 & 0\\ 0 & 0.03 & 0 & 0 & 0 & 0.1 & 0 & 0 & 0 & 0.72 & 0 & 0\\ 0 & 0 & 0.03 & 0 & 0 & 0 & 0.1 & 0 & 0 & 0 & 0.72 & 0\\ 0 & 0 & 0 & 0.03 & 0 & 0 & 0 & 0.1 & 0 & 0 & 0 & 0.72 \end{array}\right)\left(\begin{array}{c} I_{1,1}\\ I_{2,1}\\ I_{3,1}\\ I_{4,1}\\ I_{1,2}\\ I_{2,2}\\ I_{3,2}\\ I_{4,2}\\ I_{1,3}\\ I_{2,3}\\ I_{3,3}\\ I_{4,3} \end{array}\right).$$

By inverting this equation, we can obtain the intensity distributions without crosstalk:

(5) $$I_{p}^{\lambda }=C^{-1}I_{pf}^{ct}.$$

## 3. Results and Validation

### 3.1. Reconstruction Pipeline and Polarimetric Data Reduction

As described in Section 2.1, the raw image acquired by the RGB polarization camera is a spatially multiplexed mosaic, where each pixel corresponds to a specific combination of analyzer orientation and color filter. Therefore, before calculating the polarimetric quantities, the raw mosaic must be separated into full resolution analyzer intensity images for each color polarization channel.

In this work, Fourier domain demosaicking is used for this reconstruction step. This method is well suited to the periodic sampling structure of DoFP sensors and provides nearly exact reconstruction for band-limited image content. To justify this choice, Table S1 in the Supplementary Material compares Fourier interpolation with linear, cubic spline, and Makima interpolation using simulated RGB mosaic data. The results show that Fourier interpolation gives the lowest reconstruction error for smooth band-limited functions, such as Gaussian and sinusoidal patterns, whereas all methods show larger errors near discontinuities, such as rectangular functions. Therefore, Fourier demosaicking is used here as a controlled reconstruction step to obtain consistent analyzer intensity images before applying the color crosstalk correction. The objective is not to introduce a new demosaicking algorithm, but to evaluate the effect of color crosstalk correction on the reconstructed polarimetric quantities.

After Fourier domain demosaicking, 12 analyzer-dependent intensity images are obtained, corresponding to the four analyzer orientations and the three RGB camera channels. These reconstructed intensities are denoted as $I_{p,f}^{ct}$, where (p) indicates analyzer orientation and (f) the camera color channel. The superscript ct indicates that, after demosaicking, the intensities may still contain spectral crosstalk color.

The calibrated color crosstalk model is described in Section 2.2 is then applied to the 12 reconstructed color polarization channels using the matrix defined in Equation (4). This step converts the reconstructed RGB camera channel intensities into wavelength-dependent analyzer intensities associated with red, green, and blue wavelength. After this correction, the wavelength-specific analyzer intensity vector is used with the corresponding calibrated analyzer matrix (${\vec{A}}_{p}^{\lambda }$) to reconstruct the Stokes components at each wavelength.

Thus, the complete processing chain used in both simulations and experiments is: raw RGB polarization mosaic → Fourier demosaicking → $I_{p,f}^{ct}$ → color crosstalk correction → $I_{p}^{\lambda }$ → wavelength-dependent Stokes inversion → $S_{0,\lambda_{k}},S_{1,\lambda_{k}}$, $S_{2,\lambda_{k}}$ → $DoLP_{\lambda_{k}},AoLP_{\lambda_{k}},|El_{\lambda_{k}}|.$

The standard relations between Stokes vector ($S_{0,\lambda_{k}},S_{1,\lambda_{k}}$, $S_{2,\lambda_{k}}$) and the derived polarimetric quantities ($DoLP_{\lambda_{k}},AoLP_{\lambda_{k}},|El_{\lambda_{k}}|$) are as follows: the degree of linear polarization ($DoLP_{\lambda_{k}})$ and the angle of linear polarization ($AoLP_{\lambda_{k}}$) are calculated from the reconstructed linear Stokes components such as:

(6) $$DoLP_{\lambda_{k}}=\frac{\sqrt{S_{1,\lambda_{k}}^{2}+S_{2,\lambda_{k}}^{2}}}{S_{0,\lambda_{k}}}$$

(7) $$AoLP_{\lambda_{k}}=\frac{1}{2}atan2\left(S_{2,\lambda_{k}},S_{1,\lambda_{k}}\right)$$

The AoLP is an angular quantity determined by the direction of the linear Stokes vector $\left(S_{1}|S_{2}\right)$, while DoLP represents its normalized magnitude. Therefore, AoLP becomes more sensitive to noise or reconstruction errors when DoLP is low, because the linear Stokes vector has a small magnitude. For this reason, AoLP errors are interpreted together with the corresponding DoLP values in the analysis below. Since the color polarization sensor contains only linear analyzer orientations, the last circular Stokes component $S_{3,\lambda_{k}}$ is not independently measured. When the output light can be assumed to be fully polarized, an unsigned ellipticity angle is inferred from $S_{0,\lambda_{k}},S_{1,\lambda_{k}}$, $S_{2,\lambda_{k}}$ as:

(8) $$|El_{\lambda_{k}}|=\frac{1}{2}arctan\left(\frac{\sqrt{S_{0,\lambda_{k}}^{2}-S_{1,\lambda_{k}}^{2}-S_{2,\lambda_{k}}^{2}}}{\sqrt{S_{1,\lambda_{k}}^{2}+S_{2,\lambda_{k}}^{2}}}\right)$$

Therefore, ∣El∣ is reported only as an inferred quantity under the fully polarized assumption, not as an independently measured circular polarization component. In Section 3.3, the method of evaluating the color crosstalk correction method and it is affecting Stokes vector components and the following derived polarimetric parameters will be explained.

### 3.2. Simulation of Color Crosstalk Effects

To evaluate and analyze the effect of the proposed method to mitigate crosstalk, and to measure the spatial polarization distribution in the three-color channels simultaneously, we simulate a scenario in which each wavelength transports different spatial polarization information. To describe the polarization state, we started from the standard Stokes parametrization using three parameters: degree of polarization (DoLP), angle of linear polarization (AoLP) and ellipticity (El). The intensity $\left(S_{0}\right)$ is assumed equal to one. In the present simulation, only linearly polarized states are considered, so the ellipticity (El) is set equal to 0 and $S_{3}=0$. Then the degree of polarization (DoLP) is equal to degree of linear polarization (DoLP). Therefore, we refer to this quantity as DoLP throughout this paper. The corresponding components of the Stokes vector will be given by:

(9) $$\left\{\begin{array}{c} S_{0}=1\\ S_{1}=DoLP*cos\left(2El\right)*cos\left(2AoLP\right)\\ S_{2}=DoLP*cos\left(2El\right)*sin\left(2AoLP\right)\\ S_{3}=DoLP*sin\left(2El\right) \end{array}\right..$$

Since in the simulation, the DoLP will be varied between 0.5 and 1, the simulated polarization distribution is linear partially polarized light. The three linear partially polarized light spatial distributions corresponding to each wavelength are defined as:

1. Red wavelength: the DoLP varies with the radial coordinate between 0.5 and 1; the AoLP varies with the angular coordinate between −180 and 180 degrees.
2. Green wavelength: the DoLP varies with the radial coordinate between 0.5 and 1; the AoLP varies with the X coordinate.
3. Blue wavelength: the DoLP varies with X coordinate between 0.5 and 1; the AoLP with Y coordinate.

The green channel is first shown as a representative worst case because the calibrated color crosstalk matrix presents the largest off-diagonal color leakage in the green channel, such as $C_{Gr}$ = 0.25 and $C_{Gb}$ = 0.19. The green channel is therefore used to illustrate the spatial behavior of the error. The same three simulation cases were also evaluated for red and blue channels. To avoid repetition, the red and blue error maps are provided in the Supplementary Material, whereas the numerical results of all channels are reported in Section 3.3.

**Figure 2:**
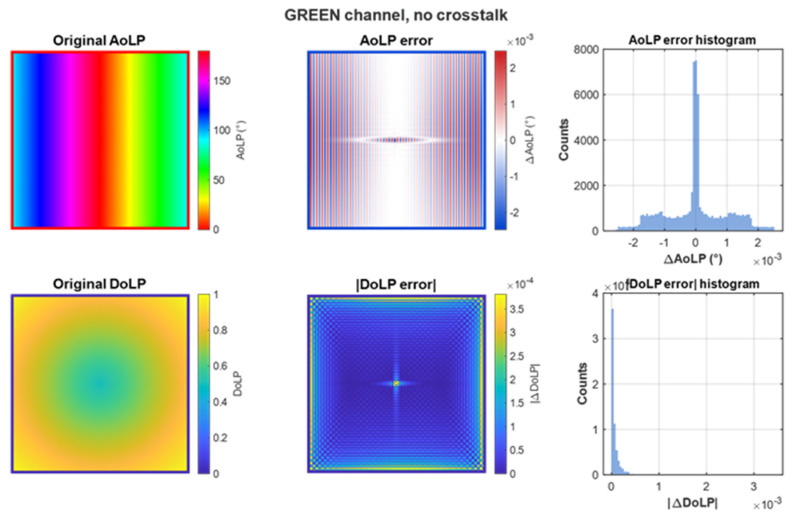
Green channel, no crosstalk. The **first row** corresponds to the AoLP and the second to the DoLP. The **left column** corresponds to the original function. The **central column** is the error (difference between the original function and the interpolated and the reconstructed one). The **right column** is a histogram of the corresponding error.

In the first simulation, we assume that there is no crosstalk, let us say, the elements outside the diagonal in Table 1 are equal to 0. Figure 2 summarizes the results obtained for the green channel. The left column presents the AoLP (first row) and DoLP (second row) parameters of the original functions. The central column shows the differences between the original and the reconstructed parameter after the intensity interpolation and polarimetric data reduction. Finally, the right column shows the histograms of these differences.

As one can see from Figure 2, the errors are almost negligible and close to the numerical precision level, with magnitudes on the order of 10^−4^. The oscillations in the AoLP errors are due to the discontinuity between the left and right borders when using Fourier Transform interpolation.

**Figure 3:**
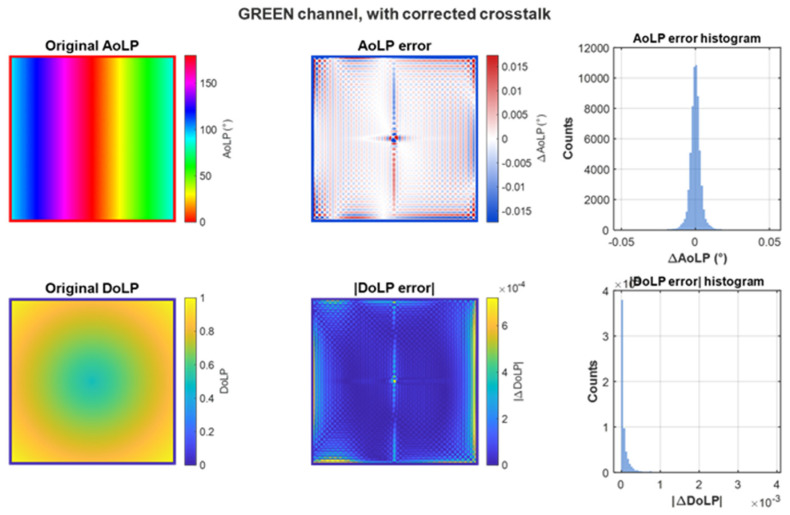
Green channel, with corrected crosstalk. The **first row** corresponds to the AoLP and the second to the DoLP. The **left column** corresponds to the original function. The **central column** is the error (difference between the original function and the interpolated and the reconstructed one). The **right column** is a histogram of the corresponding error.

In the next simulation, we assume that there is crosstalk between color channels, as described in Table 1, which is then corrected using the method described in (4). The reconstruction results can be seen in Figure 3. The resulting errors are of the same order of magnitude as those obtained in the absence of crosstalk, indicating an accurate performance of the method proposed for crosstalk correction.

**Figure 4:**
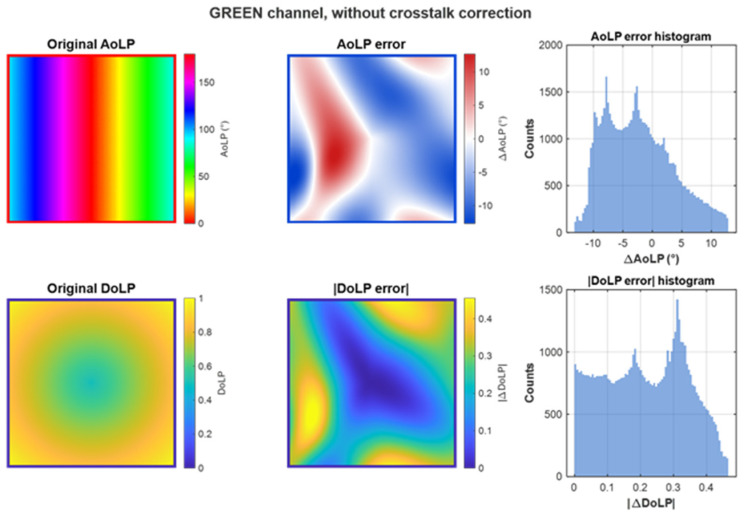
Green channel, with non-corrected crosstalk. The **first row** corresponds to the AoLP and the second to the DoLP. The **left column** corresponds to the original function. The **central column** is the error (difference between the original function and the interpolated and the reconstructed one). The **right column** is a histogram of the corresponding error.

Finally, we have made the simulation with crosstalk, but when it is not corrected in the reconstruction. The results are shown in Figure 4 for the green channel.

As we can see in this figure, the errors in the AoLP of the green channel are much larger than in the previous case with crosstalk correction. Note that some pixels have the AoLP error tail around 10°. The error in the DoLP could reach up to 50% on the green channel. This shows the need to correct the crosstalk to accurately measure with three wavelengths simultaneously.

In the Supplementary Material we show the results for the red and blue channels in Figures S1–S6. As we can see, the tendencies are the same as in the green channel. When the crosstalk is corrected the results with RGB illumination are almost identical to the results obtained with a single wavelength illumination. The errors, in general, are smaller for the red and blue channels because the crosstalk in these channels is smaller.

### 3.3. Quantitative RGB Comparison in Simulation

Although the green channel was selected in Figure 2, Figure 3 and Figure 4 as a representative case because it has the strongest spectral leakage, the correction was quantitatively evaluated for the three RGB channels. For each polarimetric quantity Q, the absolute error was computed with respect to the corresponding reference distribution as:

(10) $$e_{Q}^{case}(x,y)=|Q^{case}(x,y)-Q^{ref}(x,y)|$$

where *case* refers to either the reconstruction without crosstalk correction or with crosstalk correction. The median absolute error, MedAE, was then calculated. MedAE was used because it is less affected by the outliers.

In addition to the derived quantities, DoLP, AoLP, and El, we also evaluated the normalized linear Stokes vector error, and it was calculated as:

(11) $$|\Delta (s_{1},s_{2})|=\sqrt{{\left(s_{1}^{case}-s_{1}^{ref}{)}^{2}+(s_{2}^{case}-s_{2}^{ref}\right)}^{2}}$$

This metric is important because it evaluates the reconstructed linear polarization state directly, before reducing it to derived quantities. DoLP measures the magnitude of the normalized vector $\left(s_{1},s_{2}\right)$, while AoLP measures its direction. Therefore, $|\Delta (s_{1},s_{2})|$ gives a direct measure of how close the reconstructed polarization state is to the reference state. The relation between these quantities can be understood by writing the normalized linear Stokes vector in polar form:

(12) $$\left(s_{1},s_{2})=DoLP\;[cos(2AoLP),sin(2AoLP)\right]$$

For small errors, the vector error can be approximated as:

(13) $$|\Delta (s_{1},s_{2}){|}^{2}\approx (\Delta DoLP{)}^{2}+(2\;DoLP\;\Delta AoLP{)}^{2}$$

This relation shows that the normalized Stokes vector error contains both magnitude error, related to DoLP, and directional error, related to AoLP. Therefore, DoLP, AoLP, and $|\Delta (s_{1},s_{2})|$ are complementary and should be interpreted together.

**Figure 5:**
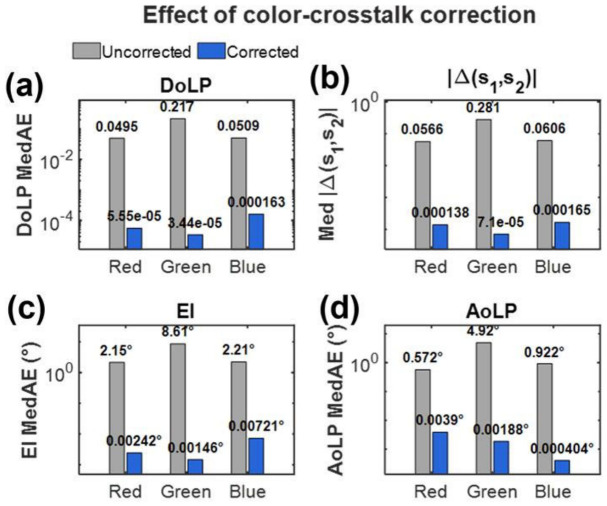
Effect of color crosstalk correction in simulation using log scale in y-axis to show the much smaller corrected errors. Panels show (**a**) DoLP MedAE, (**b**) $Med|\Delta (s_{1},s_{2})|$, (**c**) El MedAE, and (**d**) AoLP MedAE.

Figure 5 summarizes the MedAE of DoLP, $|\Delta (s_{1},s_{2})|$, El, and AoLP before and after color crosstalk correction for the red, green, and blue channels. As shown in Figure 5, the reconstruction before correction of the color crosstalk produces the largest errors in all polarimetric quantities within each color channel. The green channel shows the strongest uncorrected errors, which is consistent with the larger off-diagonal leakage terms of the calibrated crosstalk matrix. After correction, the errors decrease and close to the interpolation baseline for all three wavelengths.

This shows the need to correct the color crosstalk to have accurate measurements when three wavelengths simultaneously enter the RGB polarized image sensor.

### 3.4. Robustness to Noise and Color Crosstalk Matrix Perturbations

As shown from Equations (1)–(5), the color crosstalk correction involves the inversion of the calibrated color crosstalk matrix C, and the corrected analyzed intensities channels are obtained through an inverse operation of the calibrated crosstalk matrix C in Equation (5). In this case, the sensor noise and calibration uncertainty may propagate into the corrected analyzer intensities and the final polarimetric information. To evaluate this effect, the measured crosstalk model was written as:

(14) $$I_{pfn}^{ct}=I_{pf}^{ct}+\eta$$

where η represent additive intensity noise and $I_{pfn}^{ct}$ is the noisy image captured by the camera. The correction was then performed using a perturbed matrix:

(15) $$\tilde{C}=C+\Delta C$$

so that:

(16) $${\hat{I}}_{p}^{\lambda }={\tilde{C}}^{-1}I_{pfn}^{ct}$$

**Figure 6:**
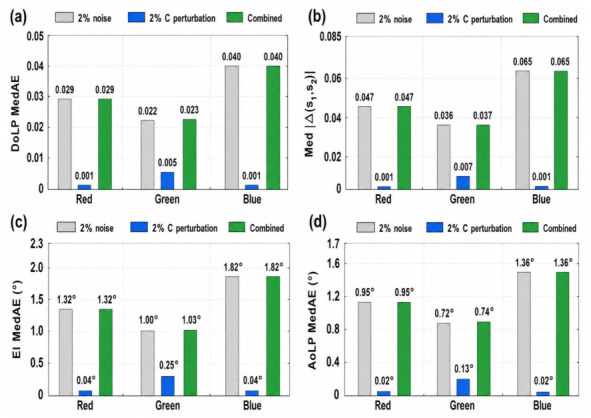
Robustness of the correction under noise and calibration perturbation. Panels show (**a**) DoLP MedAE, (**b**) $Med|\Delta (s_{1},s_{2})|$, (**c**) El MedAE, and (**d**) AoLP MedAE.

To evaluate robustness of this method, we performed a Monte Carlo analysis by adding controlled perturbations to the calibrated matrix and noise to the raw intensity values. The resulting errors were evaluated in the final polarimetric quantities. In Figure 6 the errors in the parameters defined above introduced by the noise are drawn. The gray charts correspond to the introduction of noise in the camera image. The blue charts correspond to the introduction of perturbation in the C matrix, and the green charts are obtained when both noise and perturbation are combined. The results in Figure 6 show that moderate perturbations of the crosstalk matrix produce limited degradation, indicating that the matrix inversion is numerically stable under the tested conditions. In contrast, raw intensity noise produces a stronger effect on the reconstructed polarimetric quantities, especially for angular parameters such as AoLP.

In the simulations, matrix perturbations of 2% and 5% were tested, together with the same raw intensity noise levels. The results indicate that the correction methods could be mainly limited by raw CCD noise, and even though we perturbed the C matric by 5% of perturbation, the errors remain small.

On the other hand, the robustness errors are not identical for all color channels. Although the same raw CCD noise level is applied, each channel has a different signal level, different color leakage coefficients in the C matrix, and a different simulated polarization distribution. This is particularly important for angular polarimetric parameters such as AoLP, because it depends on the direction of the normalized vectors ($s_{1},s_{2}$), and it is sensitivity increase when DoLP is low. So that in the analysis of this method in the experimental section, AoLP will be reported as a diagnostic angular quantity since errors are more sensitive to local reduction in DoLP.

### 3.5. Experimental Validation Under Sequential and Simultaneous Illumination

The illumination was provided by the NIJI system (BBO-BN7-MF-0-5-003_HPUV, BlueBox Optics Ltd. SWANSEA, WALES), which includes several LEDs equipped with spectral filters centered at 554, 633 and 470 nm with a bandwidth of 10 nm. Each LED can be switched on individually or simultaneously. Using a 5 mm-diameter, 2 m-long liquid light guide (BBO-BN7-LLG-520-001-IR) and a collimator (BBO-BN7-ZCOL-5-001), the sample was illuminated with unpolarized light.

The sample consists of a radial polarizer (Codixx, Codixx AG, Barlenben, Germany) with eight sectors, followed by a non-achromatic waveplate. The radial polarizer generates different linear polarization angles in each sector, while the waveplate transforms these states into wavelength-dependent elliptical polarization distributions. Because the waveplate is non-achromatic, the resulting DoLP, AoLP, and ellipticity distributions differ for the red, green, and blue wavelengths.

The images were captured using a LUCID polarimetric color camera (TRI05051-QC) equipped with a Sony IMX250MYR CMOS (Sony Group Corporation, Tokio, Japan) sensor and an Edmund Optics 35 mm focal-length lens (Stock No. #67716. Edmund Optics, Barrington, NJ, USA).

Two illumination modes were compared. First, the sample was illuminated sequentially with red, green, and blue single wavelength, providing the pure reference measurements for red, green, and blue channels. Second, the sample was illuminated simultaneously with three wavelengths, and the sensor recorded a single mosaic. The simultaneous acquisition was reconstructed both with and without applying the color crosstalk correction. For each wavelength, three cases were compared: pure reference for single wavelength, simultaneous RGB reconstruction without correction, and simultaneous RGB with correction.

We assume that the beams are fully polarized, so, by knowing (*S*_0_, *S*_1_, *S*_2_) we can compute the *S*_3_ component as

(17) $$S_{3}=\sqrt{S_{0}^{2}-S_{1}^{2}-S_{2}^{2}}.$$

and then we can evaluate the ellipticity (EL).

**Figure 7:**
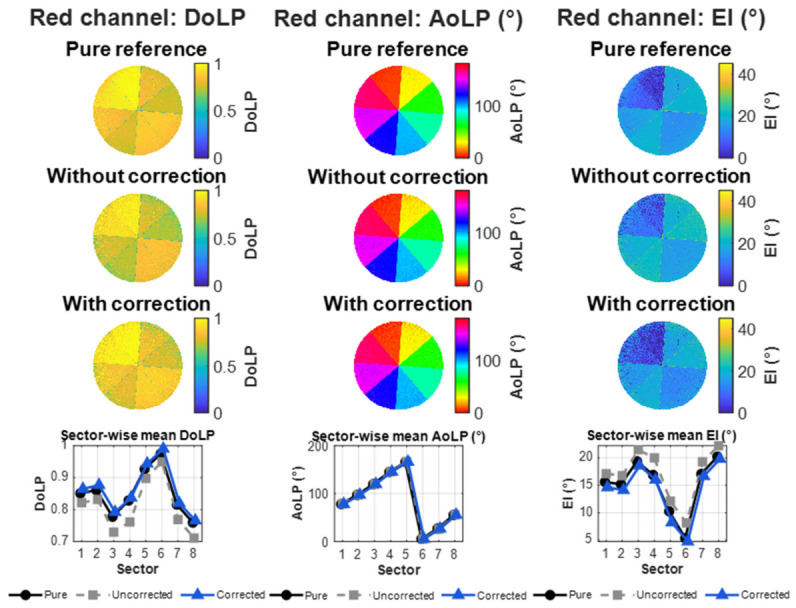
Red channel experimental validation under sequential and simultaneous illumination. Reconstructed DoLP, AoLP, and El are compared for the pure reference and simultaneous RGB illumination without and with correction. The **bottom row** shows the corresponding sector-wise mean values over the eight sectors.

**Figure 8:**
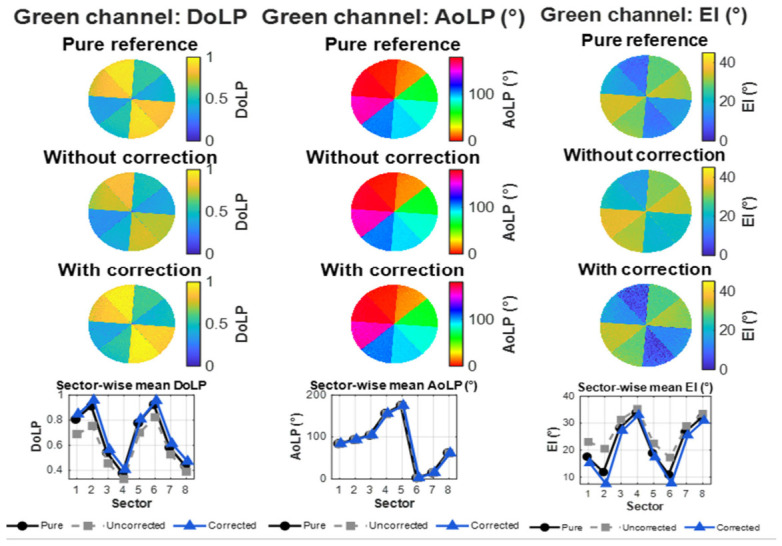
Green channel experimental validation under sequential and simultaneous illumination. Reconstructed DoLP, AoLP, and El are compared for the pure reference and simultaneous RGB illumination without and with correction. The **bottom row** shows the corresponding sector-wise mean values over the eight sectors.

**Figure 9:**
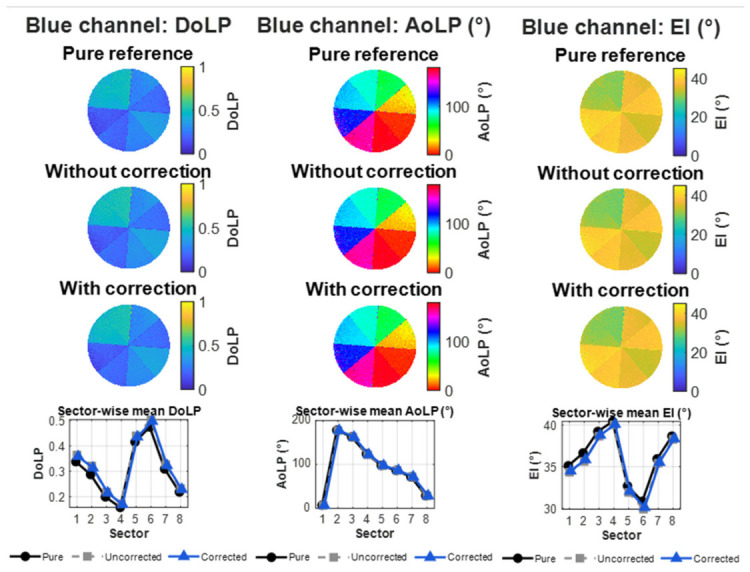
Blue channel experimental validation under sequential and simultaneous illumination. Reconstructed DoLP, AoLP, and El are compared for the pure reference and simultaneous RGB illumination without and with correction. The **bottom row** shows the corresponding sector-wise mean values over the eight sectors.

Figure 7, Figure 8 and Figure 9 visualize the reconstructed DoLP, AoLP, and EL for red, green and blue wavelengths, respectively, for the three cases of the pure single-wavelength reference, the simultaneous RGB reconstruction before correction, and after color crosstalk correction. For this comparison, the sector-wise profiles are provided. Because of the polarimetric parameters being wavelength-dependent, we have three different polarization distributions. The green channel shows widest DoLP range, approximately from 0.3 to 1, so that it exhibits a clearer correction by the method. The red channel remains mostly highly linearly polarized, with DoLP values around 0.75 to 1, so the correction appears as smaller but still shows an adjustment. The blue channel has lower DoLP values from 0.15 to 0.5 with higher El, therefore the visual improvement in blue is less pronounced.

The corrected simultaneous reconstruction agrees more closely with the single-wavelength reference than the uncorrected reconstruction, especially in the red and green channels. The blue channel shows a smaller visual improvement because of the lower color crosstalk contribution from the red and green channels are introduced. However, the mean metric can hide the error at pixel level. Therefore, in the next section, a more quantitative analysis will be presented.

For the green and red channels, the DoLP and El polarization parameters are closer to the pure reference values after correction, while the uncorrected RGB polarimetric parameters show a larger deviation. This indicates that the correction mainly compensates errors in the magnitude part of the normalized linear Stokes vector.

Since only linear analyzer states are measured, the camera directly recovers the linear part of the Stokes vector. For fully polarized elliptical states, part of the polarized energy may be carried by the circular component $S_{3}$, which is not directly measured, and the reported El is therefore calculated by applying Equation (8). Therefore, the measured DoLP decreases when the EL increases. In this sense, low DoLP does not physically imply physical depolarization of the beam, it may also indicate that a larger fraction of the fully polarized states in contained in the unmeasured circular component. As an example in these figures, The degree of linear polarization (DoLP) is shown in the first column of the figure to illustrate this effect. When the polarizer of a sector is parallel to the axis of the waveplate, the produced polarization will be linear, and the DoLP will be almost 1, while in the other cases the DoLP will decrease, with the limit case of ellipticities close to 45°, leading to the lowest DoLP values.

Each sector combined with the waveplate produces different ellipse orientations. The same results are obtained either with one or three wavelengths illuminating, showing that the proposed method is working well.

### 3.6. Quantitative Validation of the Experimental Color Crosstalk Correction

**Figure 10:**
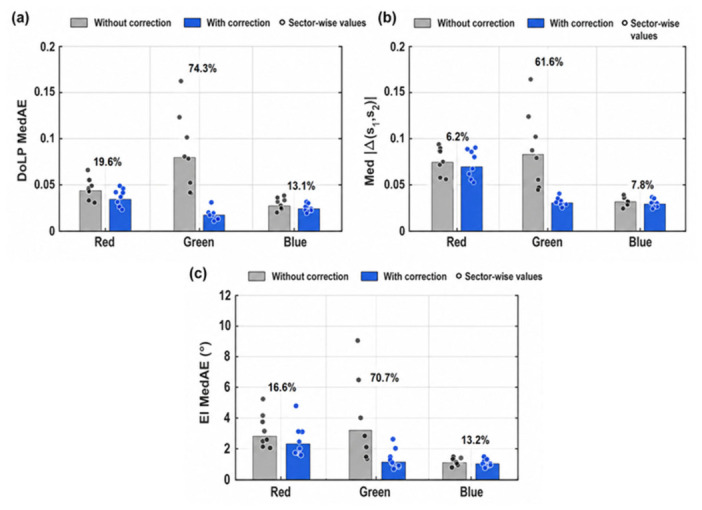
Experimental quantitative validation of the color crosstalk correction. Panels (**a**–**c**) show the median absolute error of DoLP, |Δ ($s_{1}$,$s_{2}$)| and El before and after correction. Bars show the value of the radial region and dots show the eight sectors of the radial polarizer.

To quantify the experimental performance of the correction method, the simultaneous RGB reconstruction was compared with the corresponding pure single-wavelength reference for each color channel. The comparison was performed before and after applying the color crosstalk correction. In Figure 10a–c, the bars represent the global error over the radial polarizer region, while the dots show the sector-wise errors for the eight sectors. This representation allows us to evaluate both the global correction effect and its dependence on the local polarization state.

The correction reduces the experimental errors in all three-color channels, with the strongest improvement observed in the green channel. The DoLP MedAE is reduced by 74.3%, the El MedAE by 70.7%, and the normalized Stokes vector error $|\Delta (s_{1},s_{2})|$ by 61.6%. This is consistent with the calibrated crosstalk matrix, where the green channel receives the largest leakage contribution from the other color channels. In the red and blue channels, the improvement is more moderate: the DoLP MedAE is reduced by 19.6% and 13.1%, the El MedAE by 16.6% and 13.2%, and $|\Delta (s_{1},s_{2})|$ by 6.2% and 7.8%, respectively.

The use of $|\Delta (s_{1},s_{2})|$ is important because it directly evaluates the error in the normalized linear Stokes vector. DoLP measures the magnitude of this vector, while AoLP measures its angular direction. Therefore, a reduction in DoLP error does not always imply the same reduction in $|\Delta (s_{1},s_{2})|$, because the remaining error can affect either the vector magnitude or its direction. This explains why the normalized Stokes vector error is a useful complementary metric for evaluating the correction.

Overall, the experimental results show that the proposed correction improves the RGB polarimetric reconstruction, especially in the channel most affected by spectral leakage. The remaining sector-dependent errors are consistent with the local polarization state of the radial polarizer sample and with the higher angular sensitivity of AoLP in lower-DoLP regions.

## 4. Final Remarks

The color polarization cameras give an opportunity to measure in a single shot the polarization distributions carried out by the three wavelengths. This information allows to inspect different aspects of light–matter interaction and spans the potential of single-channel illumination in polarimetric metrology. However, to efficiently operate with multichannel illumination with these devices, there are two issues that must be solved to obtain accurate measurements: the sampling of the signals and the crosstalk between the color channels. Some approaches present in the literature assume that the polarization of the different wavelengths inspected is the same, which is quite limiting in real scenarios as most natural samples present different polarimetric responses as a function of the wavelength. In this paper, we present a novel alternative that is able to be applied when measuring different wavelengths carrying out distinct polarization distribution after interacting with a sample.

Each polarization/color combination is measured in different spatial locations. Then, to accurately calculate the polarization state, interpolation is needed. We have selected the interpolation in the Fourier domain because it is the best according to the sampled signal theory for band-limited functions. Only when there are abrupt changes in intensity that produce very high frequencies or the effect of spatial crosstalk that may mix the adjacent color polarization channels before interpolation can contribute to significant errors at edges or sharp spatial transitions. However, in the study presented in this manuscript these effects are not visible.

In the color cameras, the color filters have very wide bandwidth producing color crosstalk between the color channels, i.e., the green filter also has non-zero transmission for the red and blue wavelengths. We have measured the relative transmission of each of the color filters, and with this information we have proposed a method for correcting the crosstalk, being able to determine the intensity distribution for each of the three wavelengths.

The method has been experimentally validated by measuring different samples presenting different polarization spatial distributions and for three RGB narrowband illumination wavelengths, where a current calibrated color crosstalk matrix was represented by the spectral mixing. Under broadband or white light illumination case, each RGB channel in this sensor records an integral over a continuous spectral range rather than a contribution from only three discrete wavelengths. Therefore, the color correction matrix in this method cannot directly reconstruct Stokes vector from white light data. In that case, additional spectral calibration and model would be required. The method remains applicable in principle, in case the LED central wavelengths shift, but the color crosstalk matrix should be recalibrated for actual illumination spectra used in the experiment. We have demonstrated that it is possible to measure the polarization distributions simultaneously and accurately at three different wavelengths, thereby reducing the total measurement time by a factor of three by replacing three sequential single-wavelength exposures with a single simultaneous multi-wavelength acquisition. Notably obtained results after color crosstalk correction are far superior than those without correction in terms of polarization retrieval.

Furthermore, although the method in its current form is not directly applicable to continuous spectrum measurements in the visible range, the ability to simultaneously investigate a sample across multiple spectral channels is of great interest in a wide range of applications. For instance, in the study of biological tissues, numerous works have shown that an RGB multichannel approach in the visible region can provide highly relevant information, as each wavelength interacts differently with the sample. Short wavelength illumination (e.g., blue narrowband) is typically more sensitive to superficial features, whereas longer wavelengths (e.g., red narrowband) can probe deeper structures. This enables, for example, the investigation of pathologies affecting superficial tissue layers, as well as conditions involving deeper structures due to increased penetration depth, such as certain types of cancer. Similarly, this approach is also of interest for the characterization of advanced materials, including those exhibiting microstructures, optical activity, or chiral properties. In many of these applications, it has been observed that the use of a continuous spectral distribution does not necessarily provide a significant increase in useful information, while it often leads to longer acquisition times and increased system complexity.

In all these scenarios, the method proposed in this work can offer a practical and efficient solution.

Finally, we want to note that although the proposed method is general and, in principle, applicable to real samples such as biological tissues—which often exhibit depolarization—its experimental validation in this work is limited to fully polarized systems—such as the radial polarizer and the non-achromatic waveplate—due to the use of a linear polarimetric camera. Under this constraint, depolarization and ellipticity become coupled in the measurements, preventing their independent characterization and thus limiting validation to controlled polarization states.

## Figures and Tables

**Table 1:** Relative intensities transmissions of RGB filters.

			Wavelength	
		R (630 nm)	G (554 nm)	B (470 nm)
Transmission	R-filter	1	0.04	0.01
	G-filter	0.25	1	0.19
	B-filter	0.03	0.10	0.72

## Data Availability

Data underlying the results presented in this paper may be obtained from the authors upon request.

## Supplementary Materials

The following supporting information can be downloaded at: https://www.mdpi.com/article/10.3390/s26123838/s1, Table S1. Mean reconstruction errors for the RGB DoFP mosaic using different interpolation methods. Figure S1. RED Channel, NO crosstalk. The first row corresponds to the AoLP and the second to the DoLP. The Left column corresponds to the original function. The central column is the error (difference between the original function and the interpolated and the reconstructed one). The right column is a histogram of the corresponding error. Figure S2. Red-channel simulation of color-crosstalk effects. The reconstructed AoLPand DoLPare shown for three cases: no color crosstalk, color crosstalk followed by correction, and color crosstalk without correction. For each case, the left column shows the reference distribution, the middle column shows the reconstruction error, and the right column shows the corresponding error histogram. The results illustrate that the correction reduces the crosstalk-induced error and brings the reconstruction close to the no-crosstalk baseline. Figure S3. Blue-channel simulation of color-crosstalk effects. The reconstructed AoLPand DoLPare shown for three cases: no color crosstalk, color crosstalk followed by correction, and color crosstalk without correction. For each case, the left column shows the reference distribution, the middle column shows the reconstruction error, and the right column shows the corresponding error histogram. The results confirm that the correction also improves the blue channel polarimetric reconstruction, while the remaining differences are mainly associated with interpolation and numerical effects. Figure S4. BLUE Channel, NO crosstalk. The first row corresponds to the AoLP and the second to the DoLP. The Left column corresponds to the original function. The central column is the error (difference between the original function and the interpolated and the reconstructed one). The right column is a histogram of the corresponding error. Figure S5. RED Channel, WITH corrected crosstalk. The first row corresponds to the AoLP and the second to the DoLP. The Left column corresponds to the original function. The central column is the error (difference between the original function and the interpolated and the reconstructed one). The right column is a histogram of the corresponding error. Figure S6. BLUE Channel, with NON-corrected crosstalk. The first row corresponds to the AoLP and the second to the DoLP. The Left column corresponds to the original function. The central column is the error (difference between the original function and the interpolated and the reconstructed one). The right column is a histogram of the corresponding error.

## Abbreviations

The following abbreviations are used in this manuscript:

DoFP	Division-of-Focal Plane
MPI	Microgrid Polarimetric Imaging
DoLP	Degree of Linear Polarization
AoLP	Angle of Linear Polarization
EL	Ellipticity
MedAE	Median Absolute Error
Med	Median
C	Color Crosstalk Correction Matrix
