# Supplementary material for: Color Crosstalk Correction in Linear Stokes Imaging Using a Color Polarization Camera with Simultaneous Three Wavelengths Illumination
Source: Sensors (Basel). 2026 Jun 16;26(12):3838. doi: 10.3390/s26123838 (PMC13306434; doi:10.3390/s26123838)
Supplement: Supplementary file 1 [file sensors-26-03838-s001.zip › sensors-4301155-supplementary.pdf]

Supplementary Table S1. Mean reconstruction error by functions family for the RGB DoFP mosaic.

Method	*Family*	*Mean RMSE*	*Mean MaxAbs_In4*
Linear	Rectangles	0.022	0.86
	Gaussians	$6.82x10^{-5}$	0.002
	Sine	0.012	0.038
	Sine2	0.052	0.06
Cubicspline	Rectangles	0.022471	0.87195
	Gaussians	$3.10\ x10^{-8}$	$8.40\ x10^{-7}$-07
	Sine	$9.64\ x10^{-5}$	$2.84\ x10^{-4}$-04
	Sine2	0.002	0.007
Makima	Rectangles	0.02	0.89
	Gaussians	$4.93\ x10^{-6}$	$3.19\ x10^{-4}$
	Sine	$8.59\ x10^{-4}$	0.0023
	Sine2	0.0056	0.017
FT	Rectangles	0.024	0.87
	Gaussians	$2.2398\ x10^{-8}$	$2.0942\ x10^{-7}$
	Sine	$3.4737\ x10^{-8}$	$1.3561\ x10^{-7}$
	Sine2	$2.6987\ x10^{-8}$	$7.1613\ x10^{-8}$

Table S1 summarizes the reconstruction errors obtained for the RGB DoFP mosaic using four interpolation methods: linear, cubic-spline, Makima, and Fourier-domain interpolation. The analysis is condensed over the twelve RGB color-polarization channels and grouped into four function families. The root mean square error (RMSE) is computed over the full reconstructed field, while MaxAbs denotes the maximum absolute error after excluding a 4-pixel border to reduce the influence of boundary-fill artifacts.

We have evaluated the performance of different interpolation methods: Linear, Cubic Splines, Makima and Fourier Transform. For that we have generated 12 functions with different spatial frequency contents. In matrices with 1024x1024 pixels, the following functions are generated: (1) Five sinusoidal functions with different orientations (these are band limited functions); (2) Two Gaussians with 50- and 100-pixels width that are almost band limited functions; (3) A correlated random noise with a narrow spectral band, and (4) four rectangular functions that are not band limited functions. Each one of these functions are sampled following the scheme of polarization-color given in Fig. 1 to simulate the image captured by the color polarization camera. The sinusoidal functions are band limited, but as the digital Fourier Transform assume that the functions are periodic, at the borders it could be a discontinuity if in the image do not fit an exact number of periods. The Gaussians are almost band limited because the amplitude decreases fast with the frequency. Finally, the rectangles are not band limited, their Fourier Transform are sinc functions.

As we can see, the rectangle functions give the higher errors with all the methods because of the discontinuity in the borders. For the rest of the functions the Fourier Transform based interpolation give the smaller error.

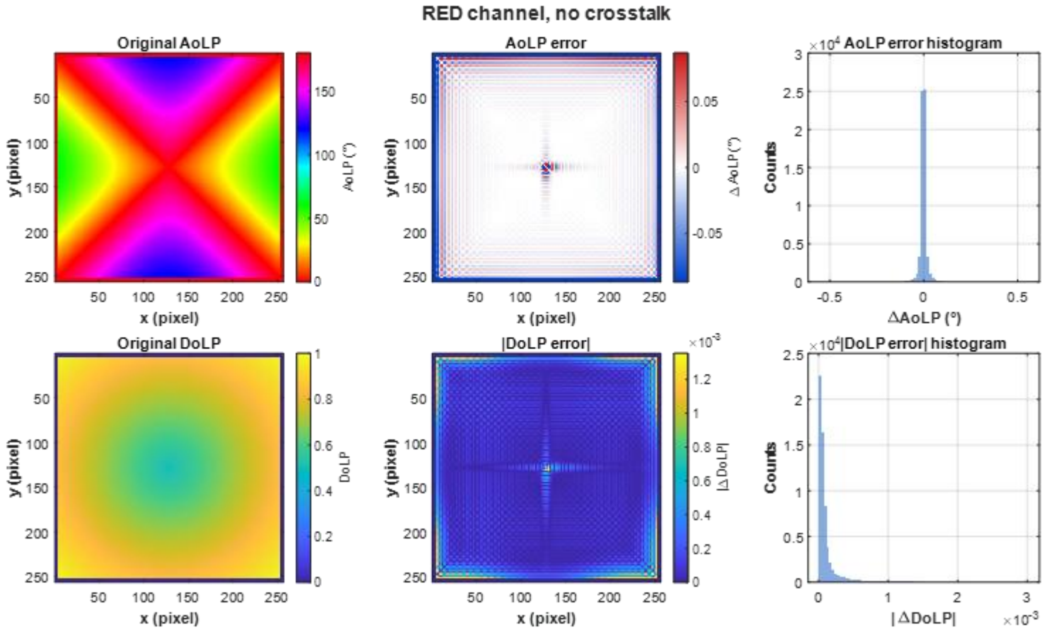

Figure S1. RED Channel, NO crosstalk. The first row corresponds to the AoLP and the second to the DoLP. The Left column corresponds to the original function. The central column is the error (difference between the original function and the interpolated and the reconstructed one). The right column is a histogram of the corresponding error.

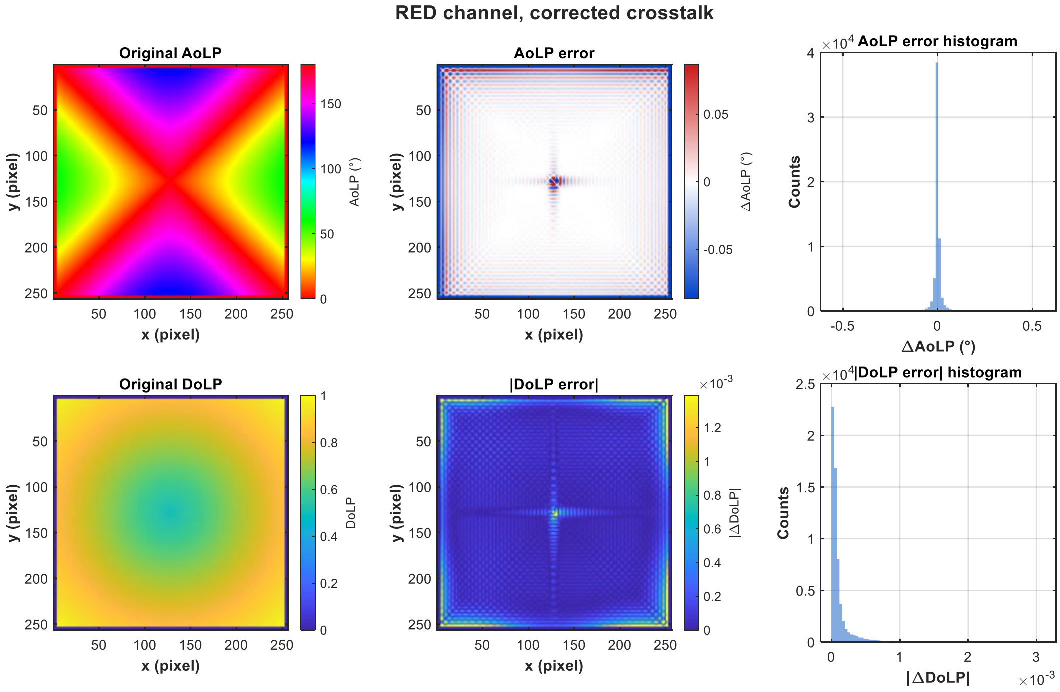

Figure S2. RED Channel, WITH corrected crosstalk. The first row corresponds to the AoLP and the second to the DoLP. The Left column corresponds to the original function. The central column is the error (difference between the original function and the interpolated and the reconstructed one). The right column is a histogram of the corresponding error.

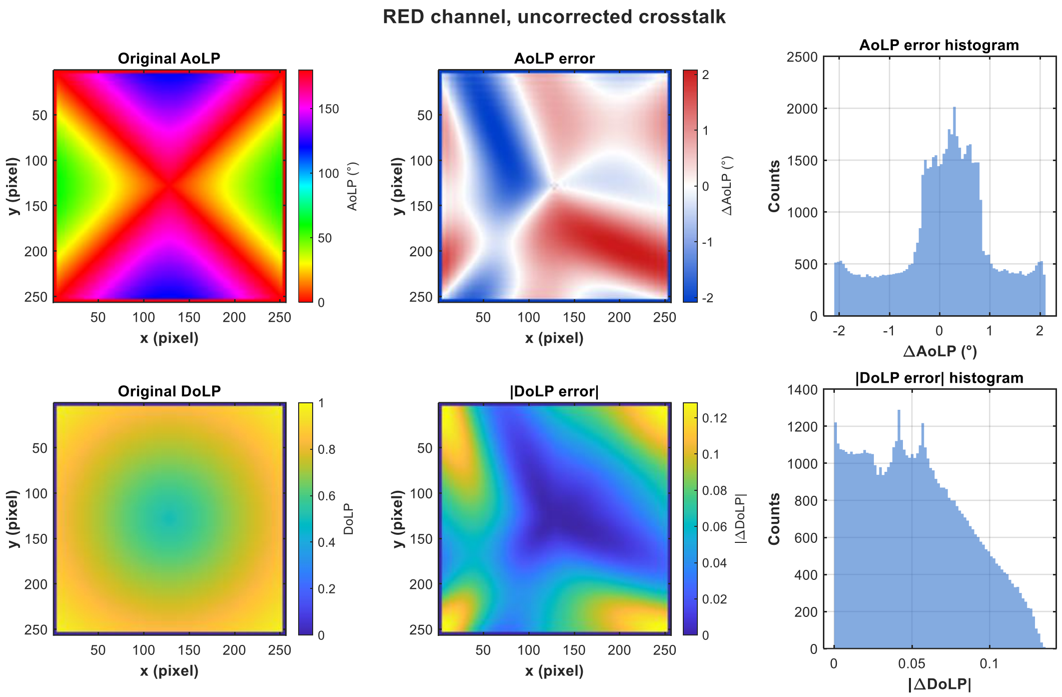

Figure S3. RED Channel, with NON-corrected crosstalk. The first row corresponds to the AoLP and the second to the DoLP. The Left column corresponds to the original function. The central column is the error (difference between the original function and the interpolated and the reconstructed one). The right column is a histogram of the corresponding error.

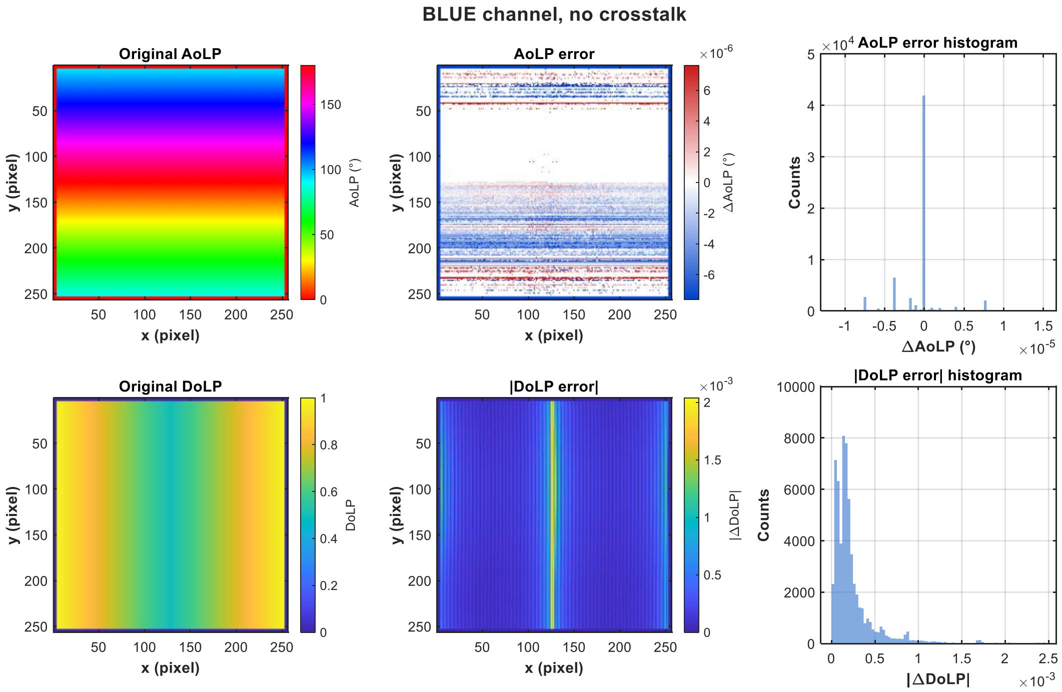

Figure S4. BLUE Channel, NO crosstalk. The first row corresponds to the AoLP and the second to the DoLP. The Left column corresponds to the original function. The central column is the error (difference between the original function and the interpolated and the reconstructed one). The right column is a histogram of the corresponding error.

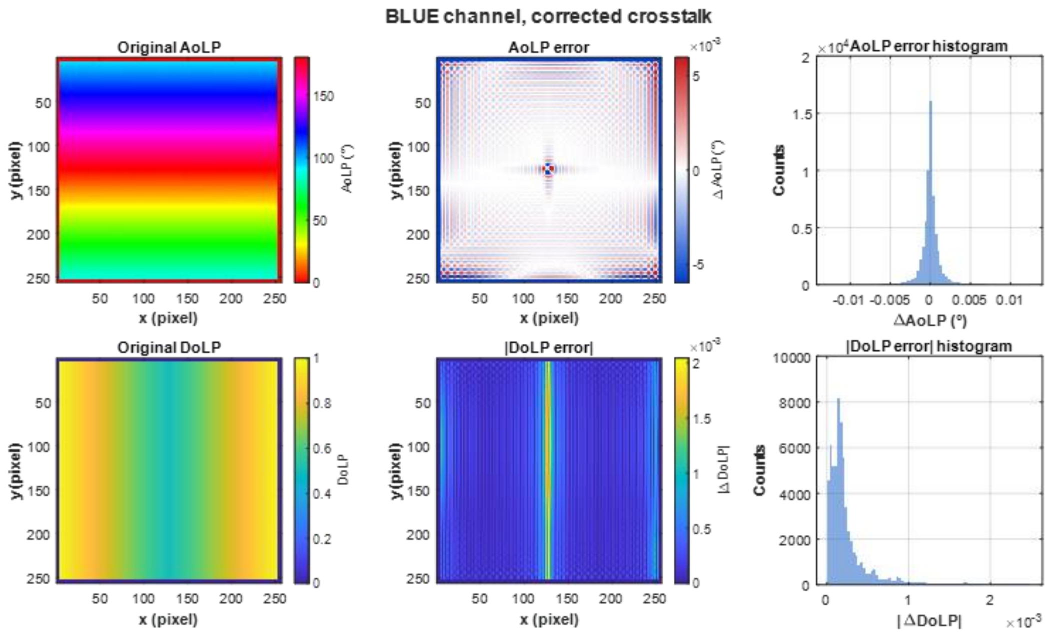

Figure S5. RED Channel, WITH corrected crosstalk. The first row corresponds to the AoLP and the second to the DoLP. The Left column corresponds to the original function. The central column is the error (difference between the original function and the interpolated and the reconstructed one). The right column is a histogram of the corresponding error.

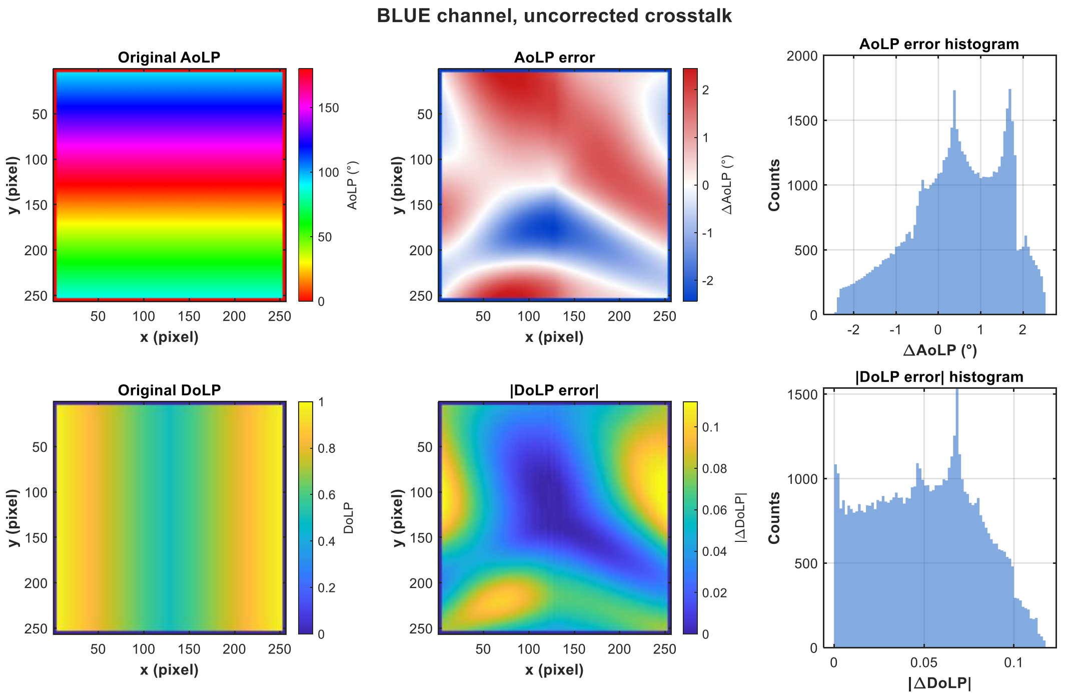

Figure S6. BLUE Channel, with NON-corrected crosstalk. The first row corresponds to the AoLP and the second to the DoLP. The Left column corresponds to the original function. The central column is the error (difference between the original function and the interpolated and the reconstructed one). The right column is a histogram of the corresponding error.

Figures S1–S6 follow the same organization that Figures 2-4 of the main text but for the red and blue channels. As we can see, the tendencies are the same as in the green channel. When the crosstalk is corrected the results with RGB illumination are almost identical to the results obtained with a single wave-length illumination. The errors, in general are smaller for the red and blue channels because the crosstalk in these channels is smaller.
